# Human brucellosis in Baringo County, Kenya: Evaluating the diagnostic kits used and identifying infecting *Brucella* species

**DOI:** 10.1371/journal.pone.0269831

**Published:** 2023-01-31

**Authors:** Nelly M. A. Waringa, Lilian W. Waiboci, Lilly Bebora, Peter W. Kinyanjui, Philemon Kosgei, Stella Kiambi, Eric Osoro

**Affiliations:** 1 Department of Biochemistry, University of Nairobi, Nairobi, Kenya; 2 Department of Veterinary Pathology, Microbiology and Parasitology, University of Nairobi, Nairobi, Kenya; 3 Department of Livestock and Fisheries, Ministry of Agriculture, Nairobi, Kenya; 4 Ministry of Health, Zoonotic Diseases Unit, Nairobi, Kenya; East Carolina University Brody School of Medicine, UNITED STATES

## Abstract

Human brucellosis diagnosis has been a challenge in *Brucella*-endemic areas. In Kenya, diagnosis is usually carried out using Febrile Brucella Antigen agglutination test (FBAT) whose performance is not well documented. This paper reports on the sensitivity and specificity of the FBAT used for brucellosis diagnosis on blood samples/serum collected in three healthcare facilities in Baringo County, Kenya, and on *Brucella* species present in the study area. The FBAT test results at the hospitals were used to guide patient management. Patients who visited the hospital’s laboratory with a clinician’s request for brucellosis testing also filled a questionnaire to assess knowledge and attitudes associated with transmission of the disease in the study area. The remaining serum samples were tested again using FBAT and Rose Bengal Plate Test (RBPT) within a month of blood collection at the University Nairobi Laboratory. The two rapid tests were then compared, with respect to brucellosis diagnostic sensitivity and specificity. To identify infecting *Brucella* species, a proportion 43% (71/166) of the blood clots were analyzed by multiplex polymerase chain reaction (PCR) using specific primers for *B*. *abortus*, *B*. *melitensis*, *B*. *ovis* and *B*. *suis*. Out of 166 serum samples tested, 26.5% (44/166) were positive using FBAT and 10.2% (17/166) positive using RBPT. The sensitivity and specificity of FBAT compared to RBPT was 76.47% and 71.19%, respectively while the positive and negative predictive values were 29.55% and 96.72%, respectively. The FBAT showed higher positivity then RBPT. The difference in sensitivity and specificity of FBAT and RBPTs was relatively low. The high FBAT positivity rate would be indication of misdiagnosis; this would lead to incorrect treatment. *Brucella abortus* was detected from 9.9% (7/71) of the blood clots tested; no other *Brucella* species were detected. Thus human brucellosis, in Baringo was mainly caused by *B*. *abortus*.

## Introduction

Brucellosis is a zoonotic disease that affects humans, domestic animals, wildlife and marine mammals [1, 2]. Wildlife and livestock are the main hosts; humans are usually accidental hosts [3, 4]. Transmission to humans is through direct contact with infected materials like afterbirth and blood, through ingestion of infected animal products such as raw milk and meat, and by inhalation of airborne agents [5, 6]. Human-to-human infection does not normally occur although there have been reports of transmissions through breast-feeding, blood transfusion and tissue transplantation [7, 8]. *Brucella melitensis* and *B*. *abortus* are the most important zoonotic *Brucella* species [5, 9, 10]. *Brucella suis* and *B*. *canis* are also zoonotic. The disease is endemic in many developing countries including countries in sub-Saharan Africa. Brucellosis is of economic importance to farmers due to losses associated with abortion, death of young ones, stillbirth, retained placenta or birth of weak calves, delayed calving, and reduction in milk yield. Brucellosis is also associated with male infertility and localized chronic conditions such as epididymitis, hygroma, arthritis and bursitis [11]. All these lead to food insecurity, poverty and loss of ability to sell animal products to international markets. In humans, brucellosis causes acute febrile illness (AFI) with undulant fever [12, 13], long-term signs and symptoms may include fatigue, recurrent fevers, arthritis, endocarditis and spondylitis and sometimes neurological symptoms [14]. Human brucellosis is often debilitating if not diagnosed early and treated correctly. It has a long duration and convalescence period, leading to economic losses in terms of treatment cost and lost man-hours. Treatment is normally expensive and extensive, lasting six weeks or longer [13].

Even though the disease has been eliminated in a number of countries, its occurrence is still on the increase in developing countries such as in sub-Saharan Africa causing a serious zoonotic risk [15, 16]. In Kenya, the disease is widely spread and endemic, especially among livestock kept by the pastoralist communities [17]; many cases have been reported in the annual reports of the Ministry of Agriculture, Livestock and Fisheries and elsewhere [18–20]. The prevalence has been shown to range from 1% -25% for bovine brucellosis, 7% -22% for caprine and ovine brucellosis and 17% -32% for human brucellosis [9, 21, 22]. Despite the growing global recognition of the economic importance of brucellosis in livestock and humans, the disease is still among the neglected, poorly understood and uncontrolled diseases [23] particularly among pastoral and agro-pastoral area in Kenya, such as Narok [18].

Opportune and correct diagnosis of brucellosis is important for proper management of the disease in humans. Isolation of the microorganism is taken as the gold standard for brucellosis diagnosis [24] in humans because it gives definitive diagnosis. However, it has serious drawbacks; it is time consuming, complicated, laborious, needs highly skilled personnel and high containment laboratory facilities (Biosafety Level 3) and has a long turnaround time [25, 26]. Furthermore, *Brucella* isolation requires a large number of viable bacteria in clinical samples, proper storage and quick delivery to the diagnostic laboratory [27]. Few studies have reported isolations of *Brucella* species from bovine milk and vaginal discharges [28]. Alternative methods used in diagnosis of human brucellosis are serological assays, including Rose Bengal Plate test, serum agglutination tests, complement fixation test, Enzyme-Linked Immunosorbent Assay and gel precipitation tests [20, 29, 30], and molecular biology techniques such as polymerase chain reaction (PCR) [31–33].

Polymerase chain reaction (PCR), although relatively rapid and accurate, is expensive and requires specialized equipment and trained staff [34]. It is currently preferred where possible and has been documented in many national and international publications [31–33]. Multiplex PCR was first developed in 1994 [25] and is still used for diagnosis and identification of *Brucella* [35]. The Multiplex PCR was named AMOS PCR using the first letters of *Brucella* species it detects, that is *B*. *abortus*, *B*. *melitensis*, *B*. *ovis and B*. *suis*. Improved AMOS PCR can detect and identify *Brucella* S19 and RB51 vaccine strains, therefore the tests differentiate *B*. *abortus* field strains from vaccine strains [36, 37]. The PCR method is independent of the disease phase [38] and can be used even when antibiotics have been administered prior to clinical specimen collection [39]. Since PCR method does not differentiate between DNA from live and dead organism [40], there is potential for detection of residual nucleic acid in patients after brucellosis has resolved; leading to false positives. PCR is fairly expensive to run and many clinical laboratories lack capacity to run it.

Brucellosis infections are mainly diagnosed using serological tests, even though it has been shown that serological results could be affected by cross-reactions with other bacteria, such as *Escherichia coli* sero-group O:157, *Yersinia enterocolitica* serovar O:9, and *Salmonella* serotypes, *Francisella tularensis*, *Pseudomonas maltophilia*, and *Vibrio cholerae* [20, 32, 41, 42]. This often leads to false-positive results and subsequently, to over-diagnosis of brucellosis, resulting in patients who are not suffering from brucellosis taking the long *Brucella* treatment unnecessarily [43–45]. Since no single serological test has been identified as the test of choice for human brucellosis diagnosis [31, 46], some studies recommended that three serological tests, be carried out to increase chances of picking seropositive samples [30, 47].

Most of the healthcare facilities in Kenya use FBAT for quick diagnosis and prompt treatment. These tests give results within a few minutes and do not require complicated infrastructure or sophisticated training. They are simple, easy to carry-out and relatively inexpensive when compared to PCR and *Brucella* isolation. Rose Bengal Plate test (RBPT) has been used as screening test for animals brucellosis but its results should be confirmed using culture or other serological test and has been recommended by some international organization for diagnosis of human brucellosis [48]. Rose Bengal Plate test has been described as the ideal test for brucellosis diagnosis in small and understaffed hospitals and laboratories, shown to be effective in the diagnosis of human brucellosis [49].

Healthcare facilities in Kenya and East African region use FBAT that are sold under different commercial names. The tests are simple and give rapid results and are affordable [6, 50, 51]. Studies have shown that commercial FBAT give high positivity rates for brucellosis cases compared to other serological tests [6, 50–52]; some of the positive cases could be due to cross-reactivity, hence presenting a misdiagnosis of the disease. Some FBAT kits have two antigens one for *Brucella abortus* and *Brucella melitensis*. However studies have demonstrated that there are no respective differences in antigenicity for the two species [35, 53, 54] because all smooth *Brucella* species share common epitopes in the O-polysaccharide (OPS) and almost all serological tests use *Brucella* antigen as whole cell [55]. It is only in detection of *B*. *ovis* and *B*. *canis* infections, rough lipopolysaccharide (RLPS) antigen is used; because the two *species* lack OPS component [56].

This study demonstrated *Brucella* positivity status of humans attending healthcare facilities in Baringo County, Kenya, using Febrile Brucella agglutination antigen test (FBAT), and Rose Bengal Plate agglutination test (RBPT), and compared the two tests with respect to sensitivity and specificity. The study also identified the *Brucella* species present in some of the samples using PCR. The study was carried out in the year 2014.

## Materials and methods

### Ethical statement

The study was approved by the Kenyatta National Hospital/University of Nairobi-Ethics and Research committee (KNH/UON-ERC, P589/11/2013). Approval was also obtained from the County Government of Baringo. Informed written consent was obtained from each enrolled participant.

### Study area

The study was carried out in Baringo County, Kenya. Baringo, which is inhabited mainly by pastoralist and agro-pastoralist communities, has human population of 666,763 (KNBS, 2019), in an area of 8,655 Km^2^. Baringo has lowland and highland regions. The lowlands are semi-arid, receive 450mm to 900mm rainfall annually, and are inhabited mainly by pastoralists. The highland areas receive 1000mm and 1500mm of rainfall annually and are inhabited mainly by agro-pastoral communities. Baringo County was chosen for this study because no similar study had been carried out in the region, *Brucella* transmission was likely due to the close interaction between the community and their livestock, known cultural practices and also patient-records in health centers had many *Brucella*-positive test results.

The three hospitals selected for the study were Marigat District Hospital (MDH) in Baringo South in the lowlands, and both Kabarnet District Hospital (KDH) in Baringo Central and Eldama Ravine District Hospital (EDH) are in the highlands. In addition to agro-pastoral communities, Eldama Ravine and Kabarnet Hospitals also serve urban/semi-urban communities.

## Study design

This was a cross-sectional laboratory based study. Sample size calculation was done using the method by Martin *et al* [57]. The confidence interval used was 95% and the assumed brucellosis prevalence was 13.7%, based on study done in Narok, Kenya a pastoralists community [58], which like Baringo is mainly inhabited by pastoralist and agro-pastoralist communities. Participants were recruited from adult patients who visited any of the study hospital’s laboratories with a clinician’s request for *Brucella* test. Inclusion criteria was patients 18 years and older and consented to participate in the study. Exclusion criteria was patients less than 18 years old and prisoners. Patients who consented were interviewed using questionnaire to collect data based on knowledge and attitudes associated with transmission of human brucellosis.

### Sample collection and handling

Five millilitres volume of blood was aseptically drawn from patients’ radial vein using 21-gauge needles, into vacutainer tubes without anticoagulant with the help of the hospital laboratory technologist. The blood was left on the bench to clot and then spun at 3000 rpm for five minutes to separate serum from the blood clot, and the serum and clot stored in separate sterile cryovials. Serum was aliquoted into two sterile cryovials, the first aliquot was used for tests requested by the attending clinicians at the hospital, which included a brucellosis test using FBAT and test results used for patient management. The second serum aliquots and blood clots were transported in a cool box (with ice packs) to the Department of Biochemistry laboratory and stored at -20°C until tested. *Brucella* tests carried out at the University’s Laboratory were FBAT, RBPT and PCR are herein reported.

### Procedure for rapid diagnostic tests

The three different FBAT kits used separately in the Baringo County hospitals were selected for this study. They were Fortress (Fortress Diagnostics Antrim, UK) used in Marigat District hospital, Plasmatec (Lab 21 Healthcare LTD, Kentford, Suffolk UK) used in Eldama Ravine District hospital and Eurocell (Euromedi Equip, Middle-UK) used in Kabarnet District hospital. The reagents and samples were brought to room temperature, two separate drops of a patient’s undiluted serum sample were placed on a white porcelain tile; to one of the drops, a drop of *B*. *abortus* antigen was added while to the other a drop of the *B*. *melitensis* antigen was added, as per the manufacturer’s instructions for rapid screening test. The procedure was repeated for the three FBAT. The serum and antigen were mixed using an applicator stick, the tile was rocked up-and-down for up to two minutes and observed for agglutination. Positive and negative serum test controls were also run. Positive reaction appeared as agglutinations/clumps; no agglutination denoted negative reaction.

### Procedure for Rose Bengal Plate Test (RBPT)

This was carried out using dyed smooth *Brucella abortus* strain 99 antigens, obtained from the Central Veterinary Investigation Laboratories, Ministry of Livestock Development, Kabete, Kenya. A drop (30μL) of patient undiluted serum was placed in a white porcelain tile to which a drop (30μL) of the antigen was added and then mixed using an applicator stick; known serum positive and negative samples were included as test controls. The tile was rocked up and down for up to four minutes. Agglutination was indicated by development of pink clumps. Agglutination was observed on positive samples. No agglutination was an indication of a negative result.

### Identification of *Brucella* species in the blood specimens

DNA was extracted from the blood clots of all the blood samples. To determine the presence and quality of extracted DNA, the DNA was resolved using electrophoresis through 0.8% agarose gel in 1 x TAE buffer stained with ethidium bromide solution (0.05mg/ml) and visualized under UV transilluminator and photographed. Due to a limitation on availability of PCR reagents, the first 71samples perceived to have higher quality DNA as determined by the gel image were amplified using AMOS PCR carried out according to Bricker and Halling [25] a positive control was extracted from *B*. *abortus* S19 vaccine strain and a negative control was extracted from a serum sample known to be negative from *Brucella*.

The PCR reaction was carried out as follows; a 20 μL reaction was prepared having 10 μL 2 × PCR buffer (50 mM Tris, 1.5 mM MgCl_2_, 10 mM KCl, 50 mM (NH_4_)_2_SO_4_, pH 8.3) (TopTaq Master Mix, QIAGEN), 6 μL double distilled sterile water, 1μL cocktail forward primers of *B*.*abortus*, *B*. *melitensis*, *B*. *ovis and B*. *suis* (Table 1) at a final concentration of 0.25 μM each, 1μL IS711 reverse primer at a final concentration of 1μM, and 2 μL DNA template for each sample. Two control reaction mixtures were included, positive control containing 2 μL *Brucella abortus* DNA and a negative control containing 2 μL double distilled water instead of specimen DNA.

**Table 1:** Polymerase chain reactions primer sequences.

Primers	Nucleotide sequence 5’ to 3’
IS711 specific -R	5’ TGC CGA TCA CTT AAG GGC CTT CAT
*B*.*abortus* specific-F	5’ GAC GAA CGG AAT TTT TCC AAT CCC
*B*. *melitensis* specific-F	5’ AAA TCG CGT CCT TGC TGG TCT GA
*B*. *ovis* specific-F	5’ CGG GTT CTG GCA CCA TCG TCG
*B*. *suis* specific-F	5’ GCG CGG TTT TCT GAA GGT TCA GG

Sources Bricker and Halling [25]

The amplification was carried out as follows, initial denaturation at 95°C for 5 min, 40 cycles of 95°C for 15 seconds, 52°C for 30 seconds, 72°C for 90 seconds and a final extension at 72°C for 5 minutes and the PCR products were stored at 4°C [59]. The amplified products were analyzed by electrophoresis through 1% agarose gel stained with ethidium bromide solution (0.05mg/ml) and visualized under UV transilluminator and photographed.

### Data analysis

Sensitivity, specificity, positive predictive value and negative predictive value for each tests and Kappa analysis were computed using epi info 7 versions 3.01 for diagnostic test evaluation [60]. Data coding for the qualitative data was done using the Statistical Package for Social Sciences (IBM SPSS Statistics 23, Chicago, USA).

## Results

### Participants

A total of 182 patients met the inclusion criteria during the period from June to August 2014, but 16 samples did not have questionnaire, therefore were excluded from the study. Of the 166 patients who consented, 116 (69.9%) were females and 50 (30.1%) were males. The patients enrolled were 84 participant from Marigat, 52 from Kabarnet, and 30 from Eldama Ravine District Hospitals (Table 2).

**Table 2:** Results of three serological tests used on the serum samples collected from Baringo Hospitals.

Hospital	FBAT	RBPT
	No. positive (%)	No. positive (%)
Kabarnet District Hospital; n = 52	9 (17.3%)	3 (5.8%)
Marigat District Hospital; n = 84	30 (35.7%)	13 (15.5%)
Eldama Ravine District Hospital; n = 30	5 (16.7%)	1(3.3%)
Overall, n = 166 (% 95% CI)	44 (26.5%)	17(10.2%)
	(20.21–33.61)	(6.26–16.14)

FBAT, Febrile *Brucella* antigen test; RBPT, Rose Bengal Plate test.

Overall, 88.6% (147/166) of the participants were aware that there is a disease although most of them referred to it using the Kiswahili term, *ugonjwa ya maziwa*. Some of the participants knew some causes of the disease, 50.6% (84/166) drinking unpasteurized (unboiled) milk and 40.4% (67/166) consumption of raw meat and/or raw blood. Some of the participants 9.0% (15/166) did not know the causes of the disease. Some of the participants 78.9% (131/166) had taken medication for the illness before enrollment to the study. This included 32.5% (54/166) who had taken traditional herbs, 21.7% (36/166) paracetamol, 18.1% (30/166) antibiotics, 6.6% (11/166) anti-malaria drugs, and 21.1% (35/166) unidentified medication. The medicine was either self-prescribed or obtained from a healthcare facility.

### General serological test results

Some serum tested positive and the positivity rate varied depending on the test used. The overall positivity rate using the FBAT was 26.5% (95% CI 20.11–34.01) (Table 2). For each sample tested the results from the three FBAT were identical. Positivity rate using RBPT was 10.2% (95%CI 6.26–16.14). In each hospital FBAT had a higher positivity rate than RBPT.

### Sensitivity and specificity and predictive values of *Brucella* diagnostic tests

The sensitivity of FBAT was 76.5%, 95% CI 52.74–90.45 and specificity was 79.2%,95% CI 71.98–84.94 the positive predictive value was 29.5%, 95% CI 28.16–44.22 and negative predictive value was 96.7% when compared to RBPT (Table 3). The agreement of FBAT and RBPT was 32.7% indicating fair agreement [61].

**Table 3:** Sensitivity, specificity and predictive values of FBAT compared to RBPT.

Febrile Brucella agglutination antigen test	Rose Bengal plate test		Total
	Number positive	Number negative	
Number positive	13	31	44
Number negative	4	118	122
Total	17	149	166
Sensitivity = 76.47%, 95%, CI 52.74–90.45 n = 17			
Specificity = 79.19%, 95%, CI 71.98–84.94 n = 149			
Positive predictive value positive = 29.55%, 95% CI 18.16–44.22 n = 44			
Negative predictive value = 96.72%, 95% CI 91.87–98.72 n = 122			
Fair agreement = 0.327, 95% CI 0.20–0.46.			

### Identification of infecting *Brucella* species

**Fig 1:**
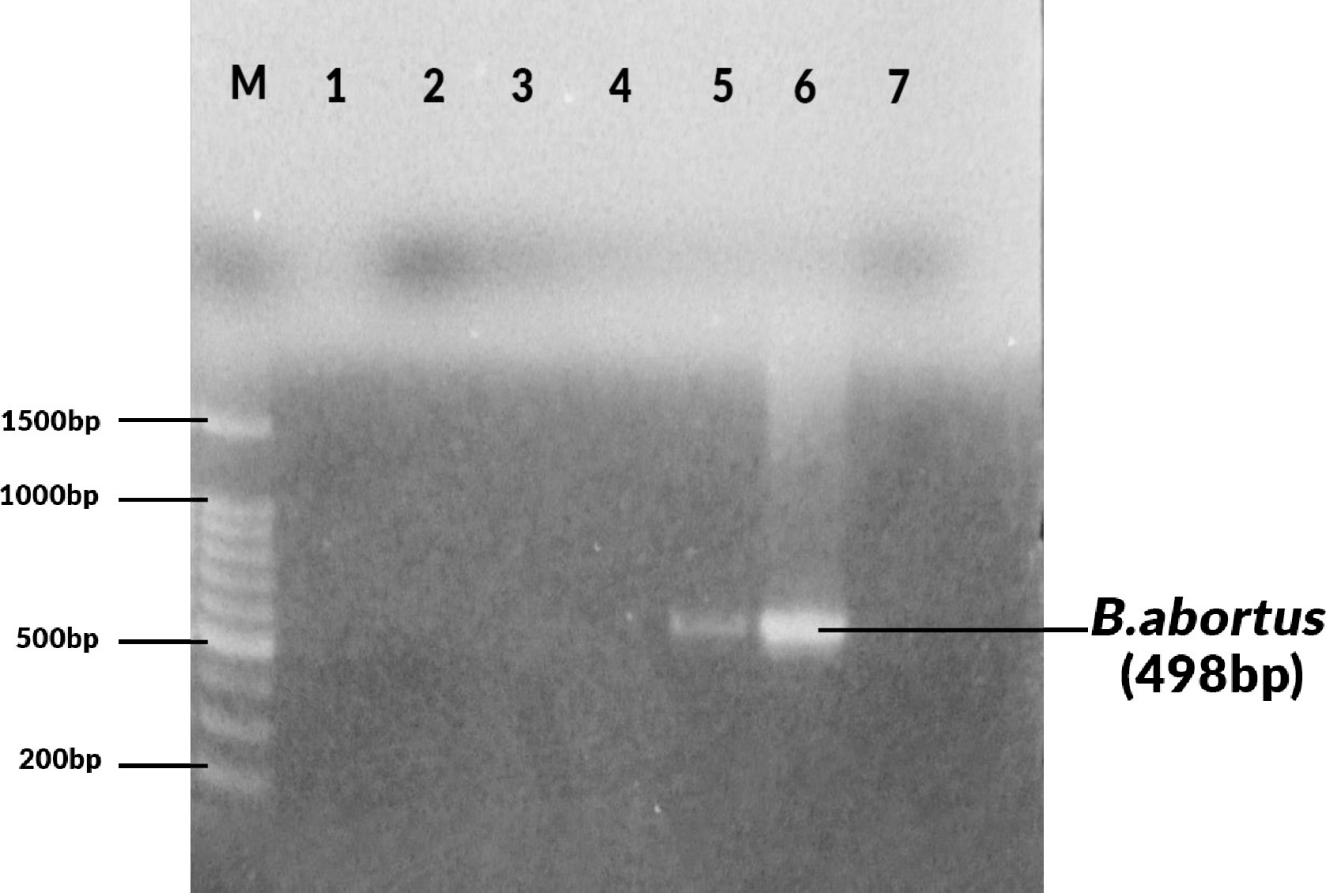
Agarose gel electrophoresis showing 100bp molecular marker (M), negative samples 1, 2, 3, 4, positive samples 5, positive control 6 (S19 *B*. *abortus*) and negative control 7 (double distilled water).

**Fig 2:**
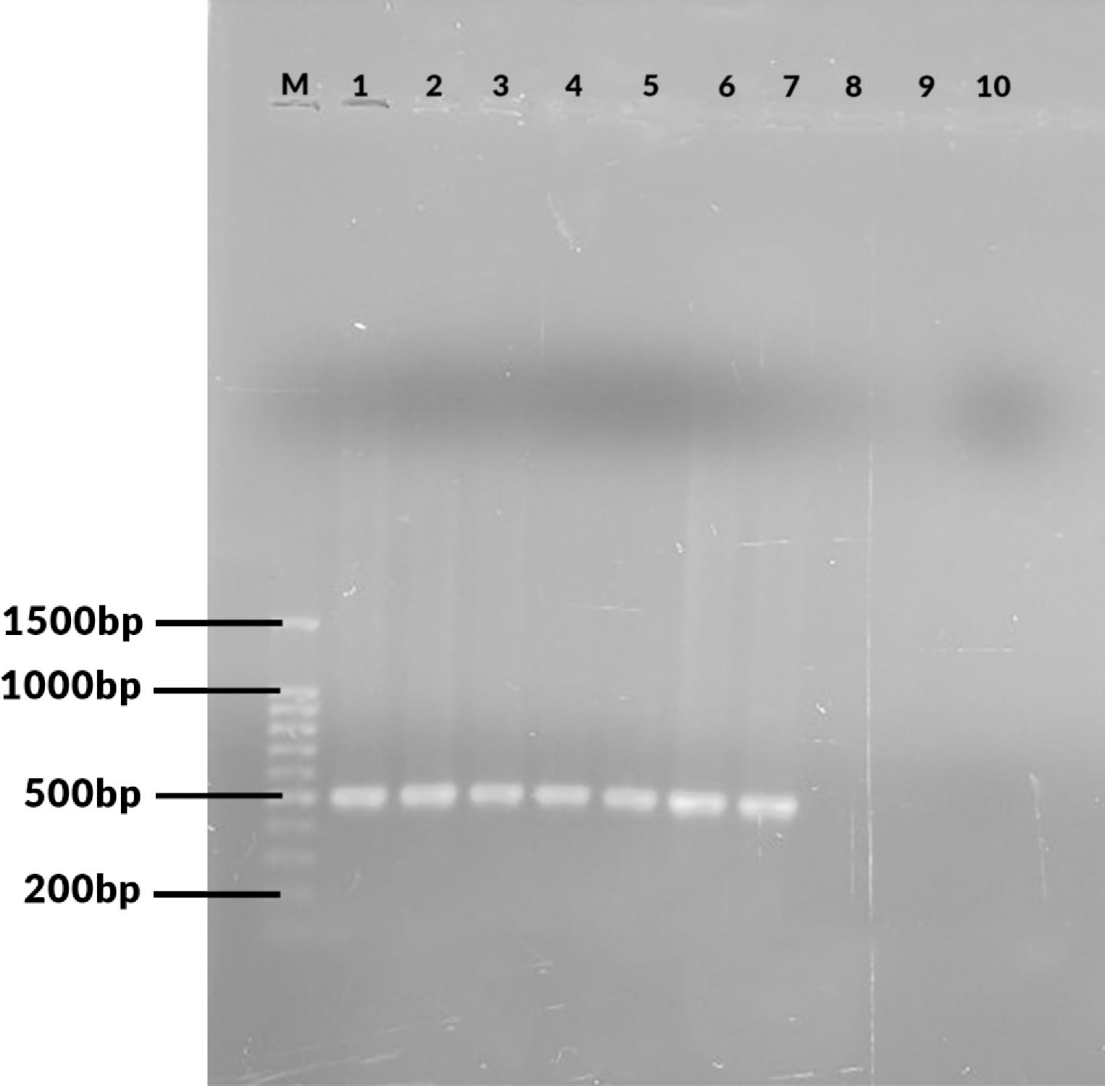
AMOS PCR, gel electrophoresis showing 100bp molecular marker (M), positive human samples 1,2,3,4,5,6,7 (*B*. *abortus* (498bp) negative human samples 8, blank well 9 and negative control 10.

Seven out of 71 (9.9%) specimens analyzed were positive for *B*. *abortus* (498 bp). No amplicons were observed that corresponded with the expected amplicon sizes for *B*. *melitensis* (731 bp), *B*. *ovis* (976 bp) or *B*. *suis* (285bp). Fig 1, shows negative samples, positive sample, positive control (S19 *B*. *abortus*) and negative control (double distilled water) and Fig 2, showing the seven positive samples (*B*. *abortus* (498 bp).

## Discussion

The study revealed that brucellosis is present in Baringo as demonstrated by presence of positive samples in the three study hospitals. This was supported by study done on livestock by Kosgei and Lokamar *et al* [62, 63]. The two tests used had varied positivity: FBAT detected the higher overall positivity of 26.5%; RBPT positivity was 10.2%. The same was the case when addressed per study hospital: FBAT consistently detected higher positivity of 35.7%, 17.3%, and 16.7% for Marigat, Kabarnet and Eldama-Ravine hospitals, respectively, than RBPT. The high percentage recorded by FBAT compared to RBPT indicated a possibility of overestimation of positivity. Since FBAT were the ones used for *Brucella* diagnosis at the study hospitals, the possible over-diagnosis of brucellosisin patients, could explain the high brucellosis cases recorded in the study hospitals (respective hospital records); some of the patients being incorrectly treated for the disease. The finding in this study agrees with a study carried out in two health facilities in Busia County in Kenya [50]; the recorded positivity rate was 19.6% using FBAT, 1% using RBT, 0.62% using serum agglutination test (SAT), 1.9% using Coombs test; also, in this study, out of the positive FBAT samples, only 3.4% were positive using the rapid IgM lateral flow immunochromatography assay (LFA). A study carried out in Ijara district hospital in Garissa in Kenya showed positivity rate of 32% using FBAT and 15.4% using real-time PCR(RT-PCR) [6]. Another study on twelve hospitals in Western Kenya showed positivity rate of 13.3% using FBAT and 1.7% using RBT [51]. In Tanzania a study on performance of four commercial FBAT showed the positivity rates ranging between 25.2% and 45.0% while that for RBT was 6.0% and 8.7% and for cELISA was 16.3% [64]. These studies demonstrated that FBATs have a tendency of overestimating brucellosis cases which mean many patients go through brucellosis treatment while actually they are not infected.

In this study, using RBPT, Kabarnet District Hospital and Eldama Ravine District Hospital both in agro-pastoralist areas, had positivity rate of 5.8% and 3.3%, respectively; these were lower compared to 7.0% to 15.3% rates reported in agro-pastoralist areas. However Marigat District Hospital positivity rate was 15.5%, which was within the range of 14.4% and 46.5% documented in a systematic review of human brucellosis in local pastoralist communities in Kenya [9]; RBPT was the test used in most of the studies. Marigat District Hospital, which has its main catchment among pastoralist communities, recorded the highest positivity of the three study hospitals (35.7%) using FBAT; this compared well with study at Ijara, Garissa [6] which is local pastoralist area.

The FBAT used in this study were supplied as two antigens, one for *B*. *abortus* and the other for *B*. *melitensis*, however, the two antigens gave identical results when tested. The sample that was positive for *B*. *abortus* was also positive for *B*. *melitensis* and the sample negative for *B*. *abortus* was also negative for *B*. *melitensis*. This confirming that, as expected that the two antigen *B*. *abortus* and *B*. *melitensis* could not be differentiated using these test kits [35, 53, 54]. The authors observed that validation of the commercial FBAT prior to use for diagnosis at the hospitals was not routinely carried out. Further, there were no specific guidelines provided by the health authorities on laboratory diagnosis for *Brucella* at the time of this study. This meant that each healthcare facility procured and used the kits that they perceived to be most cost-effective, resulting in different kits being used in different hospitals and in some cases in the same hospital.

Studies have demonstrated that FBAT produced by various manufacturers, are used widely in healthcare centres in Kenya [6, 50, 51] despite their poor performance. It has also shown that the sensitivity of these FBAT is low in the region where this study was carried. Since there are possibilities that FBAT would pick some false positives, there need to be cautions on interpretations of respective results in healthcare facilities in Kenya. For the OIE/WHO-recommended rapid test, RBPT, Diaz-Aparicio *et al* [49] have shown that cross-reactivity with *Yersinia* or any other antigens disappears with increased serum dilution; agglutination at 1:8 dilution and above was shown to indicate definite positive reaction for brucellosis. This way, the specificity of the RBPT can be greatly improved, to give tangible results; however, caution needs to be taken when dealing with low *Brucella* titres, which may have end-points at lower dilutions; in this case, one will need to also do one or two other tests to rule out the cross-reactivity with *Yersinia* or other antigens. This dilution method has now gained appraisal by a number of researchers [3, 49]; who have shown that the RBPT dilution method improves its specificity and considerably reduces the need for additional serological tests. Another point in support for RBPT is that the test is affordable and easy to run; hence it can easily be made available to healthcare facilities. Compared to other serological tests, RBPT has been shown to be exceedingly cheap; Moriyon [65] and others, compared the costing for RBPT and ELISA (which is currently the mostly used) in Spain and showed that while competitive ELISA and indirect ELISA cost 6 and 5 euros per test, respectively, RBPT costs a mere 5 cents (0.05) of an euro per test. Correct and timely diagnosis of brucellosis will save patients who are not suffering from brucellosis the long and expensive treatment.

*Brucella abortus* was the species identified from the tested samples in this study; this was demonstrated by positive PCR amplification of *B*. *abortus* (498bp) in seven patient specimens. Similar results were obtained from a study which was concurrently run on livestock in the area; *B*. *abortus* was detected in samples collected from cattle and sheep [62]. The fact that *B*. *abortus*, was isolated from cattle and patients in the study area confirm the source for the human disease. This transmission of brucellosis from animals to humans is documented [20, 22, 62]. There is, therefore, need to educate the Baringo residents on how to safeguard themselves from getting infected; from the questionnaire data, some people in Baringo County did not know what causes the disease thus they could be easily get infected.

The study, however, was not without limitations; first, being a cross-sectional hospital-based; the results cannot be generalized to the general population. Second, the method of recruitment of participants was based on a clinician’s recommendation and differences in clinicians’ understanding/view on brucellosis may have led to differences in patients’ recruitment. Third, due to the exclusion of children (<18 years) the study cannot be generalized to the general population. It has been shown that there is an increased probability of brucellosis in individuals of lower age who herd cattle, sheep and goats [66], activities that are associated with pastoralism. Fourth, most of the patients sampled were females. This is possibly due to differences in health-seeking behavior between males and females; a study in Kibera, Kenya, showed that males preferred self-treatment rather than visiting healthcare facilities [67]; this study reported more females seeking treatment than males as more women were recruited during the study period. A randomized community based study needs to be carried out in future to address this limitation. The fifth RBPT was used to compare FBAT, though recommended by OIE as a better screening test the test also has challenges. The sixth PCR was not done on all the DNA samples extracted therefore we cannot rule out the presence of other *Brucella* species.

In conclusion, this study shows that human brucellosis is present in Baringo County, the healthcare facilities use FBAT for brucellosis diagnosis; the FBAT are not validated by the hospitals before use, and the sensitivity of these FBAT is low compared to RBPT. There is need to validate FBAT used and the kits should have strong positive, weak positive and negative control samples for use in validation of the kits, if they are still to be used in healthcare facilities or their use should be discontinued. The study recommends the use of Rose Bengal Plate test (RBPT) as it is the one recommended by OIE and WHO; it is validated and is relatively inexpensive and easy to use. Further, the study has shown that *B*. *abortus* causes human brucellosis in Baringo County. Individual in these areas should be sensitized on control and preventive measures to stop animal to human transmission.

## Supporting information

S1 File (DOCX)
