# Supplementary material for: Human brucellosis in Baringo County, Kenya: Evaluating the diagnostic kits used and identifying infecting Brucella species
Source: PLoS One. 2023 Jan 31;18(1):e0269831. doi: 10.1371/journal.pone.0269831 (PMC9888686; doi:10.1371/journal.pone.0269831)
Supplement: S1 File — (DOCX) [file pone.0269831.s001.docx]

**Supporting Information**

**Human brucellosis in Baringo County, Kenya: evaluating the diagnostic kits used and identifying infecting *Brucella* species**

Nelly M. A.Waringa^1*^, Lilian W. Waiboci^1^, Lilly Bebora^2^, Peter W. Kinyanjui^1^, Philemon Kosgei^2^, Stella Kiambi^3^, Eric Osoro^4^

^1^ University of Nairobi, Department of Biochemistry, Nairobi, Kenya

^2^ University of Nairobi, Department of Veterinary Pathology, Microbiology and Parasitology, Nairobi, Kenya

^3^Ministry of Agriculture, Department of Livestock and Fisheries, Nairobi, Kenya

^4^ Ministry of Health, Zoonotic Diseases Unit, Nairobi, Kenya

^*^Corresponding Author: email: [nelly.akinyi@uonbi.ac.ke](mailto:nelly.akinyi@uonbi.ac.ke) (NW)

**Results**

**
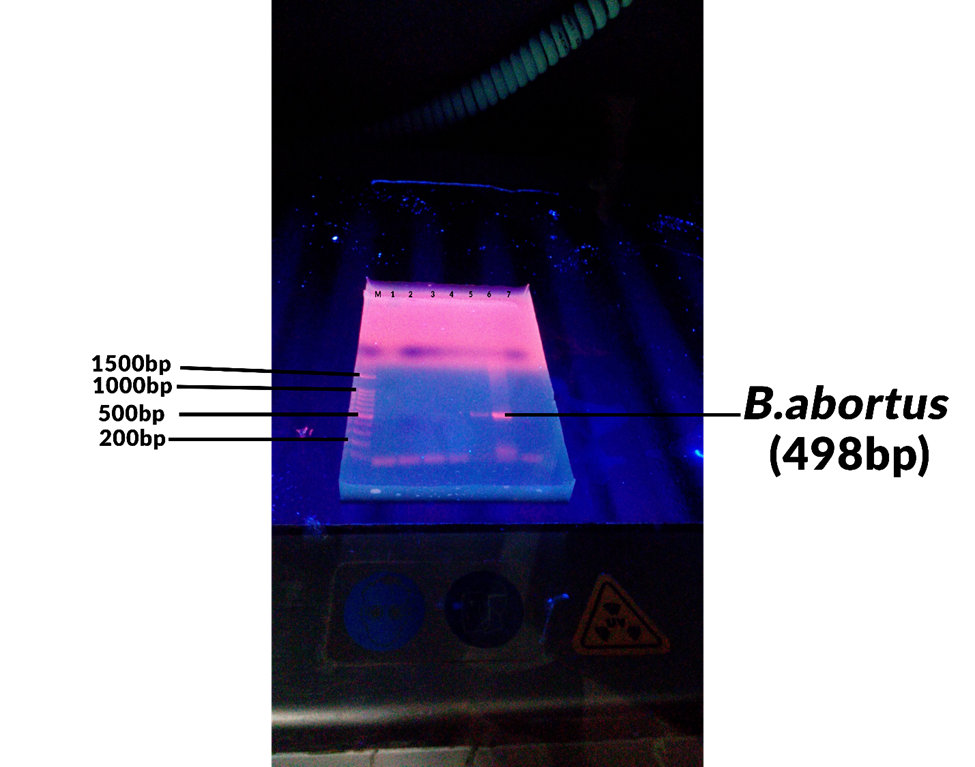
**

**S1 Fig** : Original gel doc image of agarose gel electrophoresis showing 100bp molecular marker (M), negative samples 1, 2, 3, 4, positive samples 5, positive control 6 (S19 *B. abortus* ) and negative control 7 (double distilled water). The image was captured using smartphone while the gel is viewed in gel doc because camera of gel doc was faulty.

**
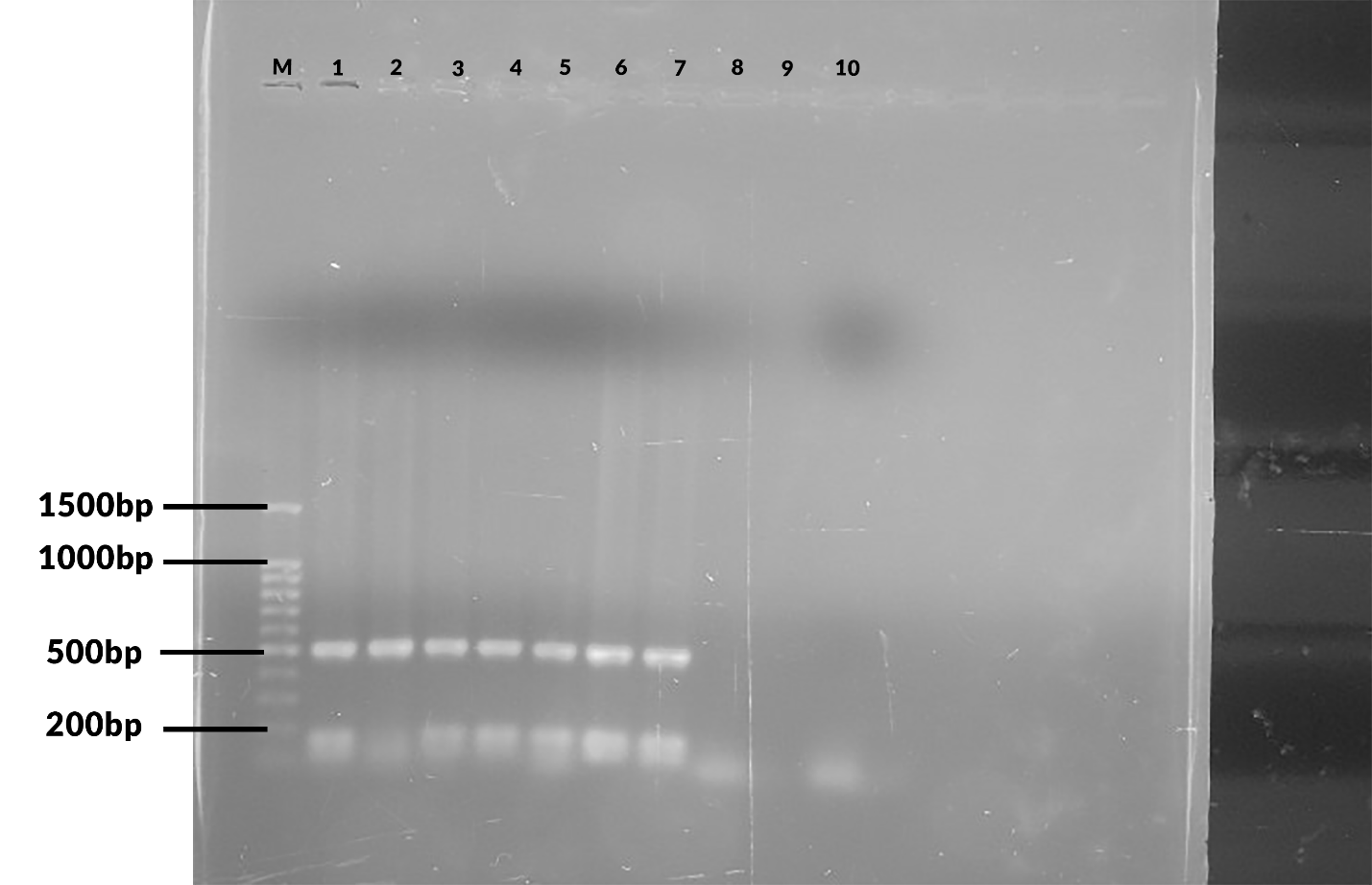
**

**S2 Fig** Original gel doc image of AMOS PCR, gel electrophoresis showing 100bp molecular marker (M), positive human samples 1,2,3,4,5,6,7 ( *B. abortus* (498bp) negative human samples 8 , blank well 9 and negative control 10. The image was captured using gel doc.

**RESULTS for the FBAT, RBPT and PCR**

**Kabarnet District Hospital**

|  | **Sample No.** | **Fortress**  **Kit** | **Plasmatec**  **Kit** | **Eurocell**  **Kit** | **Rose Bengal test** | **PCR**  **Assay** |
| --- | --- | --- | --- | --- | --- | --- |
| **1** | K001 | negative | negative | negative | negative | negative |
| **2** | K003 | **positive** | **positive** | **positive** | negative | negative |
| **3** | K004 | negative | negative | negative | negative | negative |
| **4** | K005 | negative | negative | negative | negative | negative |
| **5** | K007 | negative | negative | negative | negative | negative |
| **6** | K008 | **positive** | **positive** | **positive** | negative | negative |
| **7** | K009 | negative | negative | negative | **positive** | negative |
| **8** | K010 | **positive** | **positive** | **positive** | negative | negative |
| **9** | K011 | negative | negative | negative | negative | negative |
| **10** | K012 | **positive** | **positive** | **positive** | negative | **positive** |
| **11** | K013 | negative | negative | negative | negative | negative |
| **12** | K014 | negative | negative | negative | negative | ND |
| **13** | K015 | negative | negative | negative | negative | ND |
| **14** | K016 | negative | negative | negative | negative | negative |
| **15** | K017 | negative | negative | negative | negative | ND |
| **16** | K018 | **positive** | **positive** | **positive** | negative | ND |
| **17** | K019 | negative | negative | negative | negative | ND |
| **18** | K020 | negative | negative | negative | negative | negative |
| **19** | K021 | negative | negative | negative | negative | ND |
| **20** | K022 | negative | negative | negative | negative | ND |
| **21** | K023 | negative | negative | negative | negative | negative |
| **22** | K024 | negative | negative | negative | negative | negative |
| **23** | K025 | negative | negative | negative | negative | ND |
| **24** | K026 | negative | negative | negative | negative | ND |
| **25** | K027 | negative | negative | negative | negative | ND |
| **26** | K028 | negative | negative | negative | negative | ND |
| **27** | K029 | negative | negative | negative | negative | ND |
| **28** | K030 | negative | negative | negative | negative | negative |
| **29** | K031 | negative | negative | negative | negative | ND |
| **30** | K032 | negative | negative | negative | negative | ND |
| **31** | K033 | negative | negative | negative | negative | ND |
| **32** | K034 | negative | negative | negative | negative | ND |
| **33** | K036 | negative | negative | negative | negative | ND |
| **34** | K037 | negative | negative | negative | negative | ND |
| **35** | K040 | negative | negative | negative | negative | negative |
| **36** | K041 | negative | negative | negative | negative | ND |
| **37** | K042 | negative | negative | negative | negative | ND |
| **38** | K043 | negative | negative | negative | negative | ND |
| **39** | K044 | **positive** | **positive** | **positive** | **positive** | negative |
| **40** | K045 | negative | negative | negative | negative | ND |
| **41** | K046 | negative | negative | negative | negative | ND |
| **42** | K047 | negative | negative | negative | negative | ND |
| **43** | K048 | negative | negative | negative | negative | ND |
| **44** | K051 | negative | negative | negative | negative | negative |
| **45** | K052 | negative | negative | negative | negative | negative |
| **46** | K053 | negative | negative | negative | negative | ND |
| **47** | K054 | negative | negative | negative | negative | ND |
| **48** | K055 | negative | negative | negative | **positive** | negative |
| **49** | K056 | negative | negative | negative | negative | ND |
| **50** | K057 | **positive** | **positive** | **positive** | negative | negative |
| **51** | K060 | **positive** | **positive** | **positive** | negative | negative |
| **52** | K061 | **Positive** | **Positive** | **Positive** | negative | negative |

**Eldama Ravine District Hospital**

|  | **Sample No.** | **Fortress**  **Kit** | **Plasmatec**  **Kit** | **Eurocell**  **Kit** | **Rose Bengal test** | **PCR**  **Assay** |
| --- | --- | --- | --- | --- | --- | --- |
| **1** | E1 | negative | negative | negative | negative | ND |
| **2** | E2 | negative | negative | negative | negative | ND |
| **3** | E3 | negative | negative | negative | negative | ND |
| **4** | E4 | negative | negative | negative | negative | negative |
| **5** | E5 | **positive** | **positive** | **positive** | negative | ND |
| **6** | E6 | **positive** | **positive** | **positive** | negative | negative |
| **7** | E7 | negative | negative | negative | negative | ND |
| **8** | E8 | negative | negative | negative | negative | negative |
| **9** | E9 | **positive** | **positive** | **positive** | negative | ND |
| **10** | E11 | negative | negative | negative | negative | ND |
| **11** | E12 | negative | negative | negative | negative | ND |
| **12** | E13 | negative | negative | negative | negative | ND |
| **13** | E14 | **positive** | **positive** | **positive** | **positive** | ND |
| **14** | E15 | negative | negative | negative | negative | ND |
| **15** | E16 | negative | negative | negative | negative | ND |
| **16** | E17 | negative | negative | negative | negative | ND |
| **17** | E18 | negative | negative | negative | negative | ND |
| **18** | E19 | negative | negative | negative | negative | ND |
| **19** | E20 | negative | negative | negative | negative | ND |
| **20** | E21 | negative | negative | negative | negative | negative |
| **21** | E22 | negative | negative | negative | negative | ND |
| **22** | E23 | negative | negative | negative | negative | ND |
| **23** | E24 | negative | negative | negative | negative | ND |
| **24** | E25 | negative | negative | negative | negative | ND |
| **25** | E26 | negative | negative | negative | negative | ND |
| **26** | E27 | negative | negative | negative | negative | negative |
| **27** | E28 | negative | negative | negative | negative | negative |
| **28** | E29 | negative | negative | negative | negative | ND |
| **29** | E30 | negative | negative | negative | negative | negative |
| **30** | E31 | negative | negative | negative | negative | negative |
| **31** | E32 | **positive** | **positive** | **positive** | negative | negative |
| **32** | E33 | negative | negative | negative | negative | negative |
| **33** | E34 | negative | negative | negative | negative | negative |
| **34** | E36 | negative | negative | negative | negative | ND |
| **35** | E37 | negative | negative | negative | negative | ND |
| **36** | E38 | negative | negative | negative | negative | ND |

**Marigat District Hospital**

|  | **Sample No.** | **Fortress Kit** | **Plasmatec Kit** | **EurocellKit** | **Rose Bengal test** | **PCR**  **Assay** |
| --- | --- | --- | --- | --- | --- | --- |
| 1 | M1 | **positive** | **positive** | **positive** | **positive** | negative |
| 2 | M4 | negative | negative | negative | negative | ND |
| 3 | M5 | negative | negative | negative | negative | ND |
| 4 | M6 | negative | negative | negative | negative | ND |
| 5 | M7 | negative | negative | negative | negative | ND |
| 6 | M8 | **positive** | **positive** | **positive** | negative | ND |
| 7 | M9 | **positive** | **positive** | **positive** | negative | negative |
| 8 | M10 | negative | negative | negative | negative | ND |
| 9 | M11 | **positive** | **positive** | **positive** | negative | negative |
| 10 | M12 | **positive** | **positive** | **positive** | negative | negative |
| 11 | M13 | negative | negative | negative | **positive** | ND |
| 12 | M14 | negative | negative | negative | negative | ND |
| 13 | M15 | **positive** | **positive** | **positive** | negative | negative |
| 14 | M16 | negative | negative | negative | negative | ND |
| 15 | M17 | negative | negative | negative | negative | ND |
| 16 | M18 | negative | negative | negative | negative | ND |
| 17 | M19 | negative | negative | negative | negative | ND |
| 18 | M20 | negative | negative | negative | negative | ND |
| 19 | M21 | **positive** | **positive** | **positive** | negative | ND |
| 20 | M22 | negative | negative | negative | negative | ND |
| 21 | M23 | **positive** | **positive** | **positive** | negative | **positive** |
| 22 | M24 | negative | negative | negative | negative | ND |
| 23 | M25 | negative | negative | negative | negative | ND |
| 24 | M26 | **positive** | **positive** | **positive** | **positive** | negative |
| 25 | M27 | negative | negative | negative | negative | ND |
| 26 | M28 | negative | negative | negative | negative | ND |
| 27 | M29 | negative | negative | negative | negative | ND |
| 28 | M30 | negative | negative | negative | negative | ND |
| 29 | M31 | **positive** | **positive** | **positive** | **positive** | ND |
| 30 | M32 | negative | negative | negative | negative | ND |
| 31 | M33 | negative | negative | negative | negative | ND |
| 32 | M34 | **positive** | **positive** | **positive** | negative | ND |
| 33 | M35 | **positive** | **positive** | **positive** | negative | **positive** |
| 34 | M36 | **positive** | **positive** | **positive** | negative | negative |
| 35 | M37 | negative | negative | negative | negative | negative |
| 36 | M38 | **positive** | **positive** | **positive** | negative | negative |
| 37 | M39 | negative | negative | negative | negative | negative |
| 38 | M40 | negative | negative | negative | negative | ND |
| 39 | M41 | negative | negative | negative | negative | ND |
| 40 | M42 | negative | negative | negative | negative | ND |
| 41 | M43 | negative | negative | negative | negative | ND |
| 42 | M44 | **positive** | **positive** | **positive** | negative | negative |
| 43 | M45 | negative | negative | negative | negative | ND |
| 44 | M46 | negative | negative | negative | negative | ND |
| 45 | M47 | negative | negative | negative | negative | ND |
| 46 | M48 | negative | negative | negative | negative | ND |
| 47 | M49 | **positive** | **positive** | **positive** | **positive** | negative |
| 48 | M50 | negative | negative | negative | negative | ND |
| 49 | M51 | negative | negative | negative | **positive** | negative |
| 50 | M52 | negative | negative | negative | **positive** | ND |
| 51 | M53 | negative | negative | negative | negative | ND |
| 52 | M54 | negative | negative | negative | negative | ND |
| 53 | M55 | **positive** | **positive** | **positive** | negative | negative |
| 54 | M56 | negative | negative | negative | negative | negative |
| 55 | M56 | negative | negative | negative | negative | ND |
| 56 | M57 | negative | negative | negative | negative | ND |
| 57 | M58 | negative | negative | negative | negative | negative |
| 58 | M59 | negative | negative | negative | negative | ND |
| 59 | M60 | **positive** | **positive** | **positive** | negative | negative |
| 60 | M61 | **positive** | **positive** | **positive** | **positive** | negative |
| 61 | M62 | negative | negative | negative | negative | negative |
| 62 | M66 | negative | negative | negative | negative | ND |
| 63 | M67 | **positive** | **positive** | **positive** | negative | negative |
| 64 | M68 | negative | negative | negative | negative | negative |
| 65 | M69 | **positive** | **positive** | **positive** | **positive** | **positive** |
| 66 | M70 | negative | negative | negative | negative | ND |
| 67 | M71 | **positive** | **positive** | **positive** | negative | **positive** |
| 68 | M72 | **positive** | **positive** | **positive** | negative | ND |
| 69 | M73 | **positive** | **positive** | **positive** | negative | negative |
| 70 | M74 | negative | negative | negative | negative | ND |
| 71 | M75 | negative | negative | negative | negative | negative |
| 72 | M76 | negative | negative | negative | negative | negative |
| 73 | M77 | **positive** | **positive** | **positive** | negative | negative |
| 74 | M78 | **positive** | **positive** | **positive** | negative | ND |
| 75 | M79 | **positive** | **positive** | **positive** | negative | negative |
| 76 | M80 | negative | negative | negative | **positive** | ND |
| 77 | M81 | negative | negative | negative | negative | ND |
| 78 | M82 | **positive** | **positive** | **positive** | **positive** | **positive** |
| 79 | M83 | negative | negative | negative | negative | ND |
| 80 | M84 | negative | negative | negative | negative | ND |
| 81 | M85 | **positive** | **positive** | **positive** | **positive** | **positive** |
| 82 | M86 | **positive** | **positive** | **positive** | **positive** | negative |
| 83 | M87 | negative | negative | negative | negative | negative |
| 84 | M88 | negative | negative | negative | negative | negative |

FBAT, Febrile Brucella antigen test; RBPT, Rose Bengal Plate test; PCR, Polymerase chain reaction; ND, PCR was not done.
